# 

**Published:** 1919-03

**Authors:** 


### ANNOUNCEMENTS

Michigan State Dental Society, will hold its annual meeting in Detroit, April 7 to 12, inclusive dates. The first three days will be devoted to the regular exercises of the State Society, and the last three days to a post-graduate course under the direction of the Detroit Clinic Club. Extensive preparations are being made for an extraordinarily good meeting.

West Virginia State Dental Society, will meet in Wheeling, April 9 to 11, inclusive. A good meeting is assured and ethical dentists from other states are welcome.

Kentucky State Dental Association. The Fiftieth Anniversary—Jubilee Meeting—of the Kentucky State Dental Association will be held at Louisville, Ky., June 9-10-11-12, 1919. A post-graduate course of unusual interest has been planned. Address all correspondence to W. M. Randall, Secretary, Louisville, Ky.

Texas Dental Society meets April 21-24.

### PREPAREDNESS LEAGUE NOTES AND NEWS

Communication from the President.—Every day it is becoming more apparent that many of our profession who left well-established and hard-won practices are returning to find nearly everything swept away down to the lowest rung of the ladder which, through honest toil and travail, had long been passed in the upward climb.

An urgent call for help goes out from the President to every member of the League to give our brothers the fullest measure of cooperation right away. *Tomorrow* will not do. *It must be done today.*

Go to each returned dental corps officer in your neighborhood and ascertain his circumstances. Call all dentists together surrounding his location and get them to send patients to him whenever possible. You can well afford to make this sacrifice. Make it a point also, to acquaint the public of his return to practice and of his need of support. You will be surprised at the assistance you can render in thus discharging a positive obligation.

It is distressing to report that undue advantage is being taken of our boys in this way and we sincerely trust our members will do all they can to reestablish them in that which justly belongs to them. He who takes undue advantage of these conditions does not merit professional relationship with his confreres.

You will learn in due time that these men have sacrificed more than we at home were aware of, and we would emphasize the truth that he who attempts to gain through their misfortunes must suffer eventual defeat.

The President earnestly requests all returning dental corps officers to communicate with him that such assistance as is possible may be rendered.

Our Future.—The League is gradually becoming adjusted to the new conditions and its officers are planning to develop into a broad organization of international character devoted to public service work and such activities as come within the rightful field of our energies.

Post-War Course of the League.—The second course given at New York from February 3 to 8, inclusive, proved to be an intellectual feast of the rarest quality. It would be impossible to give an adequate idea of its value to those who were fortunate enough to enjoy it. It was an unqualified success!

The demand for a special course in Post-War Oral Surgery and Prosthesis is now established and the League will be glad to correspond with all who wish to take it, with a view of bringing it within reach. We hope to carry it to every large city in this country.

Red Cross Home Service Work.—General cooperation of League officers and members is being experienced throughout the country by the Red Cross Home Service Section. This most worthy object of giving needy families of our soldiers dental treatment should be taken part in by every loyal dentist.

We would especially urge your care of the families of those who made the supreme sacrifice. We have a solemn duty to perform toward all such, especially those who are handicapped by lack of means, and we must keep constantly in mind that our work is not yet done. Our slogan must be, "*Carry On.*"—J. W. Beach, *President.*

---

### THE VICTORY-LIBERTY LOAN

So far Uncle Sam has made a runaway race of it, despite the handicaps of his start. He went into it absolutely without training. Wholly unprepared. And the long track ahead was a tangle of obstacles that only a thoroughbred might face without having heart failure.

But Uncle Sam was a thoroughbred par excellence. Blood told as it always does and will. He took them all like a swallow on the wing.

The stake right now is all but won, for he is showing the whole world a clean pair of heels. From the dropping of the flag he has romped along easily, overhauling first one and then another of his better trained opponents.

He has topped every hurdle without a touch. Right now he is going easily, under wraps as it were, and with a little encouragement from us he will finish a winner under wraps.

Let's back him with our last dollar if need be.

Incidentally for ourselves, there's no better prize than Victory-Liberty Bonds.

P. S.—Don't sell your Liberty Bonds to the *scalpers*, who want to trade you out of perfectly safe investments to your certain loss. They are good enough for anybody to keep and will pay you a proper interest.

